# An Appreciation of Help

**Published:** 1915-11

**Authors:** 


### An Appreciation of Help.

In the October issue, a candid statement was made of what was needed to allow further improvement of this journal. A little over four years ago, a representative of the Journal solicited certain advertising and it has been solicited at reasonable intervals ever since without success. After the editorial on the Needs of the Journal was in type but before it was published, this advertising was promptly and easily secured, simply because the prospective advertiser happened to mention the matter to two physicians who, without cost even to their consciences, gave a favorable opinion as to the publicity value of the advertisement. There are many cases in which similar aid is possible and every gain, however slight contributes just so much to the strength of the Journal.

